# Physical resistance training-induced changes in lipids metabolism pathways and apoptosis in prostate

**DOI:** 10.1186/s12944-020-1195-0

**Published:** 2020-01-29

**Authors:** Giovana Rampazzo Teixeira, Leonardo Oliveira Mendes, Allice Santos Cruz Veras, Hayley Hope Allyssa Thorpe, Wagner José Fávaro, Luiz Gustavo de Almeida Chuffa, Patrícia Fernanda Felipe Pinheiro, Francisco Eduardo Martinez

**Affiliations:** 1 Department of Physiotherapy, School of Technology and Sciences, UNESP, campus of Presidente Prudente, São Paulo, SP Brazil; 2 Postgraduate Program in Movement Sciences, Sao Paulo State University-UNESP, Presidente Prudente, SP Brazil; 3 Multicenter Graduate Program in Physiological Sciences, SBFis, São Paulo State University (UNESP), Araçatuba, SP Brazil; 4 Postgraduate Program in Animal Science and Postgraduate Program in Health Sciences University of Western São Paulo-UNOESTE, Presidente Prudente, SP Brazil; 5 Department of Biomedical Sciences, Ontario Veterinary College, University of Guelph, Guelph, ON Canada; 6 Department of Structural and Functional Biology, State University of Campinas - UNICAMP, Institute of Biology, Campinas, SP Brazil; 7 Department of Anatomy, São Paulo State University, UNESP - Institute of Biosciences, Botucatu, SP Brazil

**Keywords:** Physical exercise, Apoptosis, CD36, SREBP-1, SCAP

## Abstract

**Background:**

Altered lipid metabolism is an important characteristic of neoplastic cells, with androgens and growth factors being major regulatory agents of the lipid metabolism process. We investigated the effect of physical resistance training on lipid metabolism and apoptosis in the adult Wistar rat prostate.

**Methods:**

Two experimental groups represented sedentary and physical resistance training. Three days per week for 13 weeks, rats performed jumps in water carrying a weight load strapped to their chests as part of a physical resistance exercise protocol. Two days after the last training session, rats were anesthetized and sacrificed for blood and prostate analysis.

**Results:**

Physical exercise improved feeding efficiency, decreased weight gain, regulated the serum-lipid profile, and modulated insulin-like growth factor-1 (IGF-1) and free testosterone concentration. Furthermore, upregulation of cluster of differentiation 36 (CD36), sterol regulatory element binding protein-1 (SREBP-1), sterol regulatory element-binding protein cleavage-activating protein (SCAP), and reduced lysosome membrane protein (LIMPII) expression were also observed in the blood and prostates of trained rats. Consistent with these results, caspase-3 expression was upregulating and the BCL-2/Bax index ratio was decreased in trained rats relative to sedentary animals.

**Conclusions:**

In this work, physical resistance training can alter lipid metabolism and increase markers of apoptosis in the prostate, suggesting physical resistance training as a potential novel therapeutic strategy for treating prostate cancer.

## Introduction

Prostatic diseases are the most common male malignancy worldwide and therapeutic approaches aim to increase prostate cancer cell apoptosis and reduce proliferation to prevent lesion progression. Altered lipid metabolism is an important characteristic of neoplastic cells, with androgens and growth factors being major regulatory agents of this lipid metabolism process, and physical exercise has been shown to increase the availability and oxidation of lipids [1, 2]. Prostatic diseases are largely related to lifestyle, specifically consumption of a high-fat diet, excessive consumption of ethanol, obesity, diabetes, smoking, and sedentary behavior [3–5].

Previous studies conducted in our laboratory have shown that strength or resistance training affects the prostate by promoting hormonal changes and cell turnover [6–8]. Intense concentric and eccentric muscle concentrations during resistance training elevates circulating levels of anabolic/catabolic hormones (i.e., testosterone, testosterone free, growth hormone, insulin-like growth factor-1 (IGF-1), cortisol) and cytokines [9]. Androgens act in the prostate microenvironment by androgen receptor (AR) activation and coordinating a range of transcription factors responsible for the expression of genes involved in the synthesis, transport, and metabolism of fatty acids, and cholesterol [10] modulates the recently characterized sterol regulatory element binding proteins (SREBPs). Considering that cholesterol is an essential requirement for cell growth and proliferation, it has been implicated in several different type of cancers, notably within prostate tumors [11]. However, it is unclear how changes in circulating lipid and anabolic hormones levels induced by long-term resistance training may affect prostate health, proliferation, and apoptosis.

It is well established that the maximal rate of muscle fatty acid oxidation is higher during physical exercise. Physical resistance training induces expression of the fatty acid transporter cluster of differentiation 36 (CD36) in muscles [12, 13]. Studies with pharmacological and immunologic CD-36 inhibitors have shown that the antiangiogenic signaling mediated by CD36 and thrombospondin 1 (TSP-1) is dependent on protein tyrosine kinases Fyn and Lyn, and Src kinases [14–16]. Exposure of endothelial cells to TSP-1 leads to the recruitment of Fyn to a CD36 membrane complex, and subsequent downstream activation of p38 mitogen-activated protein kinase [17]. Furthermore, antiangiogenic activity of TSP-1 is linked to epithelium apoptosis via caspase-3–like effectors [18]. CD36 signaling can lead to programmed cell death in other cell types, as showed by Rusiñol et al., [19] with hamster ovarian cells transfected with CD36. It has been proposed that fatty acid and cholesterol uptake may play a key role in proliferation and cell survive, and their oxidation during exercise may change the prostate epithelium [20, 21].

Surprisingly, little attention has been paid to the role of physical exercise as a potential regulator of fatty acid oxidation and metabolism in prostate. The aim of this study was to assess if repeated physical resistance training alters hormone and growth factor responses, and subsequently alters lipid metabolism pathways and apoptosis in the prostate. It is well established that exercise can change the metabolism and proportion of fatty acids in an organism, as originally shown by Pedersen & Febbraio [22], and resistance exercise can induce apoptosis in the rat prostate as we have previously shown [6, 7]. However, the molecular mechanisms underlying these changes have yet to be described.

## Materials and methods

### Ethics statement and biological materials

This study was approved by the Committee for Ethics in Animal Experimentation of the Institute of Biosciences/UNESP (protocol number: 83/07). All the procedures were conducted in accordance with the guidelines for experimentation with animals according to the Brazilian legislation on the scientific use of animals (Law No. 11.794, of October 8, 2008).

### Animals and experimental design

Twenty adults male Wistar rats (90 days of age; 250-260 g) were divided into two groups of 10 animals each representing a sedentary lifestyle and a physical resistance training exercise lifestyle. All animals were maintained at an average temperature of 22 ± 2 °C under a 12 h:12 h light:dark cycle.

The trained animals were habituated to aquatic training exercise for three sessions across the first experimental week. In brief, each training session occurred in a reservoir containing water at 30 °C in which animals were submerged by strapping a weight load equivalent to 50% of the rat’s total bodyweight to animal’s back. Rats were required to leap towards the water’s surface with increasing number of sets (two to four) and repetitions (five to 10); a 60 s rest occurred between each set [23, 24]. After the habituation period, the rats were submitted to physical resistance training for 13 weeks (91 days of training) and performed four sets of 10 jumps per set carrying a load equivalent to 50–70% of the animal’s bodyweight with a 60 s rest between each set. Two days after the last training session (191 days of age), the sedentary and trained rats were euthanized by decapitation, and the intermediate and distal regions of the ventral prostate were collected and processed for immunohistochemistry, Western blotting, and hormonal analysis.

### Food and liquid intake

During the experimental period, weekly consumption of water and food and changes in rat body mass were monitored. Food intake value and caloric value of ration for rodents (3 kcal/g) were used to obtain total energy consumption (TEI, kcal/day = average food consumption per day [g] × 3) and (ii) feed efficiency (FE, g/kcal = mean bodyweight gain / total TEI mean) [25]. Body weight was also measured, and the training weight load for rats in the trained group was adjusted accordingly. Relative prostate weight, used to evaluate the growth of prostate in different interventions, was determined as the ratio between absolute prostate weight and total animal bodyweight (g).

### Hormone quantification

Blood samples were collected in heparinized tubes from the cervical vessels following decapitation. Plasma was obtained by centrifugation at 1200×g for 15 min at 4 °C and samples were stored at − 20 °C until analysis by radioimmunoassay. The serum levels of glucose, total protein, triglycerides (TGs), total cholesterol, and high-density lipoprotein (HDL) cholesterol were enzymatically determined (Biochemical Kits Laborlab®, Guarulhos, SP, Brazil). The plasmatic levels of IGF-1 were measured by an immunoenzymatic method using the IGF-1 kit-OptEIA™ (Biosciences, San Jose, CA, USA). Free testosterone concentration was measured using a commercially-available radioimmunoassay kit (Diagnostic Products Corporation, Los Angeles, CA, USA).

### Immunohistochemistry of CD36, Bcl-2 and Bax

Samples of the ventral prostate obtained from five animals in each group were fixed in 10% formalin solution, embedded in paraffin, and cut into 4-mm thick sections. Immunohistochemistry used to evaluate the protein expression was previously described by Fávaro and Cagnon [26]. Prostate markers were evaluated through immunohistochemistry using anti-CD36 (L-17, Santa Cruz Biotechnology sc-5523; 1:200 dilution), anti-BCL-2 (N-19, Santa Cruz Biotechnology sc-492; 1:200 dilution), and anti-Bax (P-19, Santa Cruz Biotechnology sc-526; 1:200 dilution). Sections were also labeled for nuclei using the Harris hematoxylin method and tissue was photographed with a Zeiss Axiophot Photomicroscope (Zeiss, Hamburg, Germany). Ten fields were analyzed at random per animal at 40× magnification. The signals for CD36, Bcl-2, and Bax were quantified as a percentage of signal presence within the total field of view using Image-J (version 1.5).

### Western blot analysis

Samples of the ventral prostate obtained from five animals in each group were frozen, weighed, and homogenized in 50 mL/mg of lysis buffer. The technique used has been previously briefly described by Fávaro and Cagnon [27]. In brief, tissue slices were incubated with primary antibodies diluted in 1% BSA at 4 °C overnight. The following primary antibodies were used: anti-CD36 (L-17, Santa Cruz Biotechnology sc-5523), anti-SCAP (9D5, Santa Cruz Biotechnology sc-13,553), anti-LIMPII (D-4, Santa Cruz Biotechnology sc-55,571), anti-SREBP-1 (H-160, Santa Cruz Biotechnology sc-8984), and anti-caspase-3 (Abcam ab4051) at a dilutions of 1:500, 1:250, 1:250, 1:500, respectively; anti-β-actin (Abcam ab8227) was used as a control. An anti-goat secondary antibody (Santa Cruz Biotechnology sc-2354) was used against all primary antibodies at a 1:1000 dilution. The results are expressed as the mean of the ratio between the intensity of each band against the intensity of the β-actin band.

### Statistical analysis

Data, specifically measures of bodyweight, food consumption, biochemical parameters, plasma hormone levels, proliferative indices, and expression of caspase-3 and Bax and lipid profiling after physical training, are reported as mean ± SEM. According to the Shapiro-Wilk test, data were normally distributed. A Student’s t-test for independent samples was used between groups (Sigma Plot, version 14) and significance was determined at *p* < 0.05.

## Results

### Bodyweight, status of food consumption, and biochemical parameters

To determine the impacts of physical exercise on parameters of prostatic health, we subjected adult Wistar rats to either a sedentary lifestyle or physical resistance training for 13 weeks. Trained rats exhibited low body weight gain compared to the sedentary group (Table 1). However, food consumption, energy intake, and feed efficiency were higher in the rats submitted to resistance training. On the other hand, no significant differences were observed in relative and absolute prostate weights between the two groups.

**Table 1:** Nutritional and biochemistry parameters associated with sedentary lifestyle and physical resistance training

Biochemistry	Sedentary	Trained	*P*- value
Initial weight (g)	439.0 ± 7.37	421.0 ± 12.44	0.22
Final weight (g)	524.5 ± 14.9	526.0 ± 13.8	0.94
Body weight gain (g)	122.0 ± 15.7	90.0 ± 6.4	0.03*
Prostate weight (g)	0.895 ± 0.02	0.905 ± 0.017	0.73
Relative weight prostate (g)	0.171 ± 0.0052	0.173 ± 0.0041	0.84
Food consumption (g/day)	26.5 ± 0.3	27.3 ± 0.2	0.04*
Energy intake (kcal/day)	77.1 ± 1.2	80.1 ± 0.6	0.01*
Feed efficiency (g/Kcal)	116.0 ± 10.4	88.0 ± 9.9	0.03*
Glucose (mg/dL)	123.16 ± 7.5	123.49 ± 3.15	0.4
Triglyceride (mg/dL)	101.94 ± 9.01	137.86 ± 12.45	0.01*
Total protein (g/dL)	6.76 ± 0.36	7.93 ± 0.31	0.01*
Cholesterol (mg/dL)	70.31 ± 5.23	64.6 ± 8.0	0.05*
HDL (mg/dL)	18.4 ± 3.3	21.34 ± 8.98	0.05*
LDL (mg/dL)	31.75 ± 3.92	34.65 ± 7.49	0.07
VLDL (mg/dL)	20.38 ± 1.80	27.57 ± 2.49	0.01*
Triglyceride/HDL (mg/dL)	7.34 ± 1.10	8.60 ± 1.96	0.64

Values are expressed as mean ± SEM, *n* = 10. *indicates a significant statistical difference between the groups (*p* < 0.05), as determined using an independent samples Student’s t-test

As shown in Table 1, differences in feed consumption and FE were both observed between the two groups. Increases in TGs, total cholesterol, VLDL, LDL, HDL, and total protein concentrations were also observed following resistance training. In addition, we compared plasma glucose levels after resistance exercise to investigate whether training was associated with reduced glucose concentration. Interestingly, there was no significant difference in plasmatic glucose levels between groups (Table 1).

**Fig. 1:**
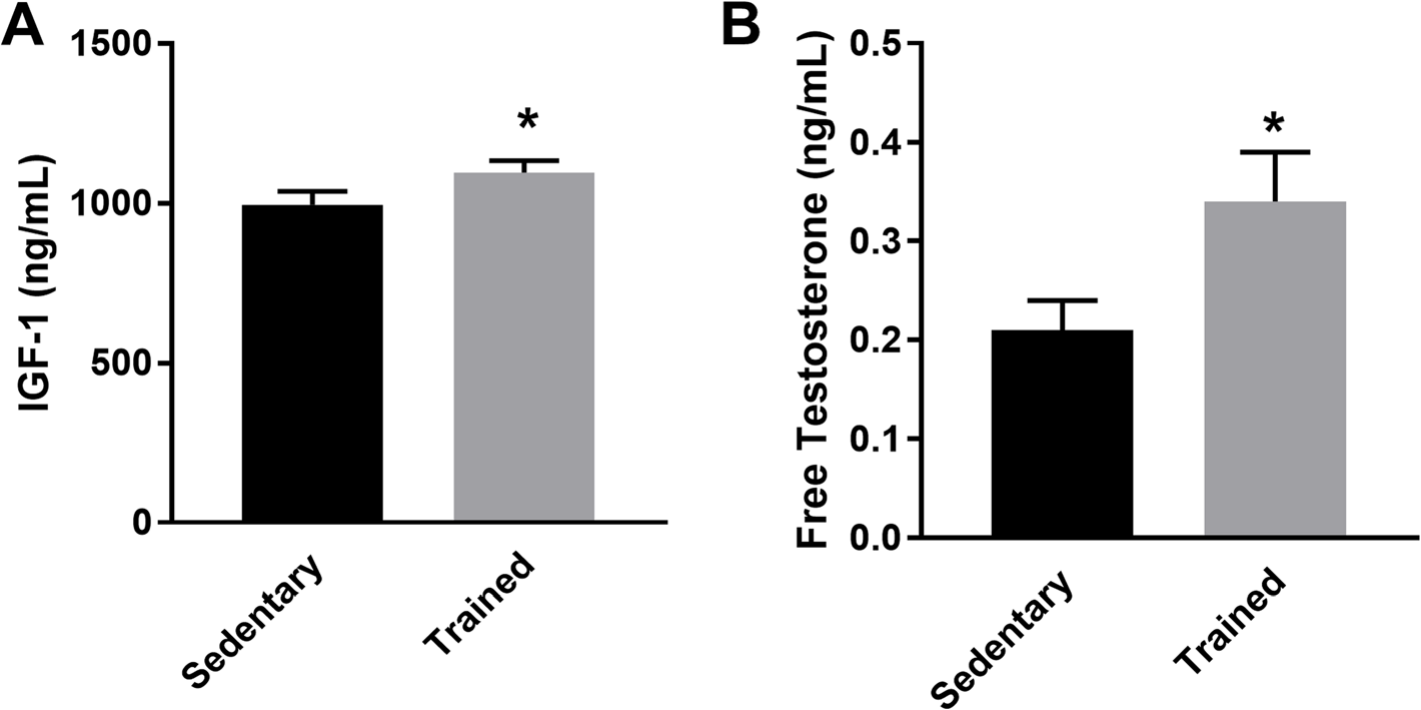
Hormone levels evaluated from blood samples harvest after 13 weeks of sedentary lifestyle or resistance training. **a** IGF-1 plasma levels (ng/dL); **b** Free testosterone levels (pc/mL). Values are expressed as mean ± SEM (*n* = 10); *p < 0.05

To investigate how physical training affects lipolysis and energy substrate mobilization/utilization, we performed biochemical tests on blood samples to determine hormonal levels after 13 weeks of resistance training. The resistance training increased plasma levels of IGF-1 (Fig. 1a) and free testosterone, indicating that resistance exercise modulates hormone expression (Fig. 1b).

### Expression of BCL-2 and Bax

**Fig. 2:**
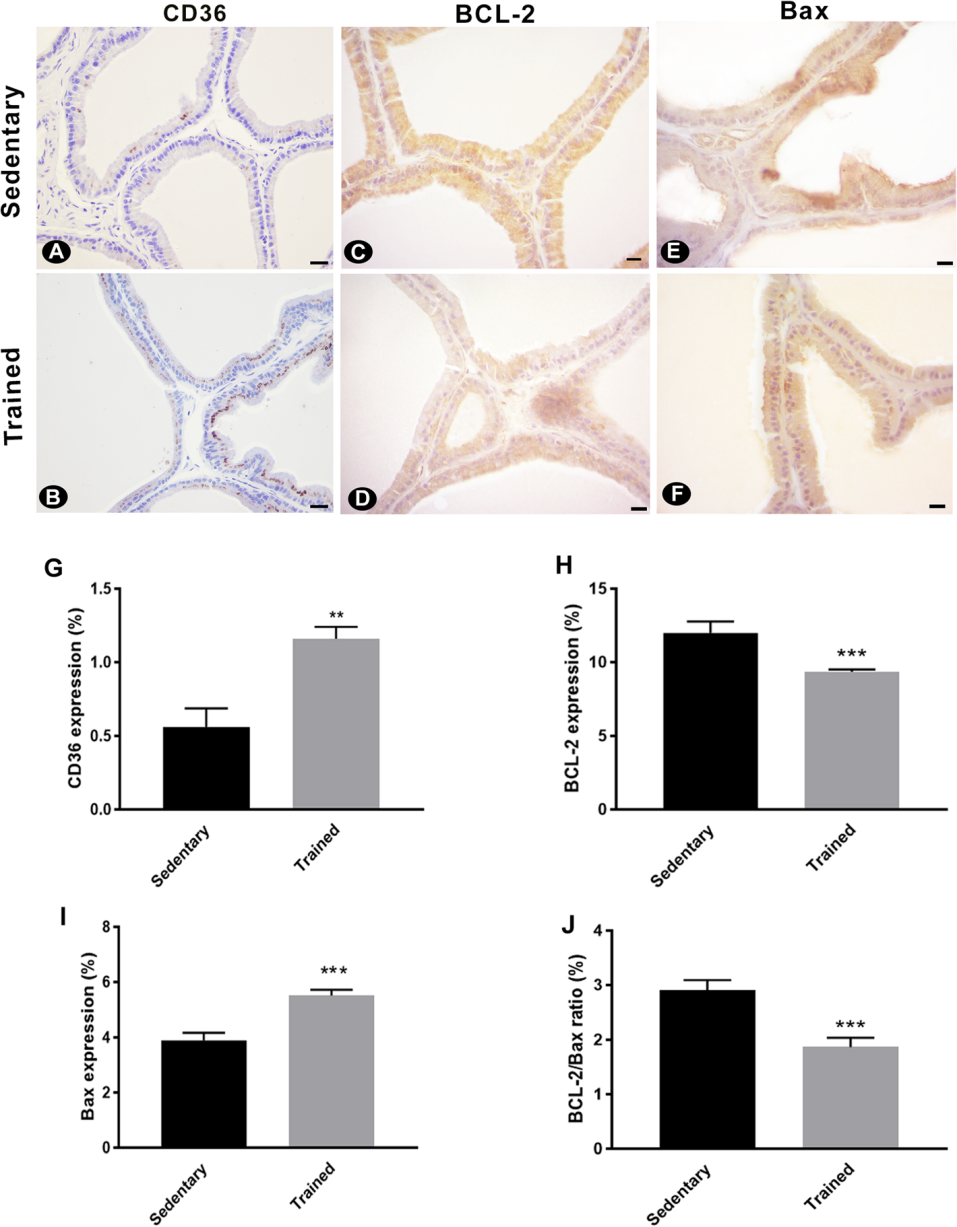
Immunohistochemical analysis of the ventral prostate following 13 weeks of sedentary lifestyle or resistance training. Representatives images of CD36 (**a-b**), BCL-2 (**c-d**), and Bax (**e-f**) expression in the prostates of sedentary and trained animals are shown. Expression of (**g**) CD36, (**h**) BCL-2, (**i**) Bax, and (**j**) the expression ratio of Bcl-2/Bax were quantified. Values are expressed as mean ± SEM (*n* = 10); **p < 0,001, ***p < 0,0001; scale bar = 20 μm

To evaluate the effects of resistance training on prostatic apoptosis and proliferation, the ventral prostate was harvested from rats following training or sedentary lifestyle and immunolabelled for BCL-2, Bax, and caspase-3. BCL-2 detection decreased in the trained rats (Fig. 2c, d, h), whereas Bax expression indices were higher when compared with the sedentary group (Fig. 2e, f, i). The BCL-2/Bax ratio was also higher for sedentary rats when compared to the trained group (Fig. 2j).

### Lipid profile and caspase-3 after physical training

**Fig. 3:**
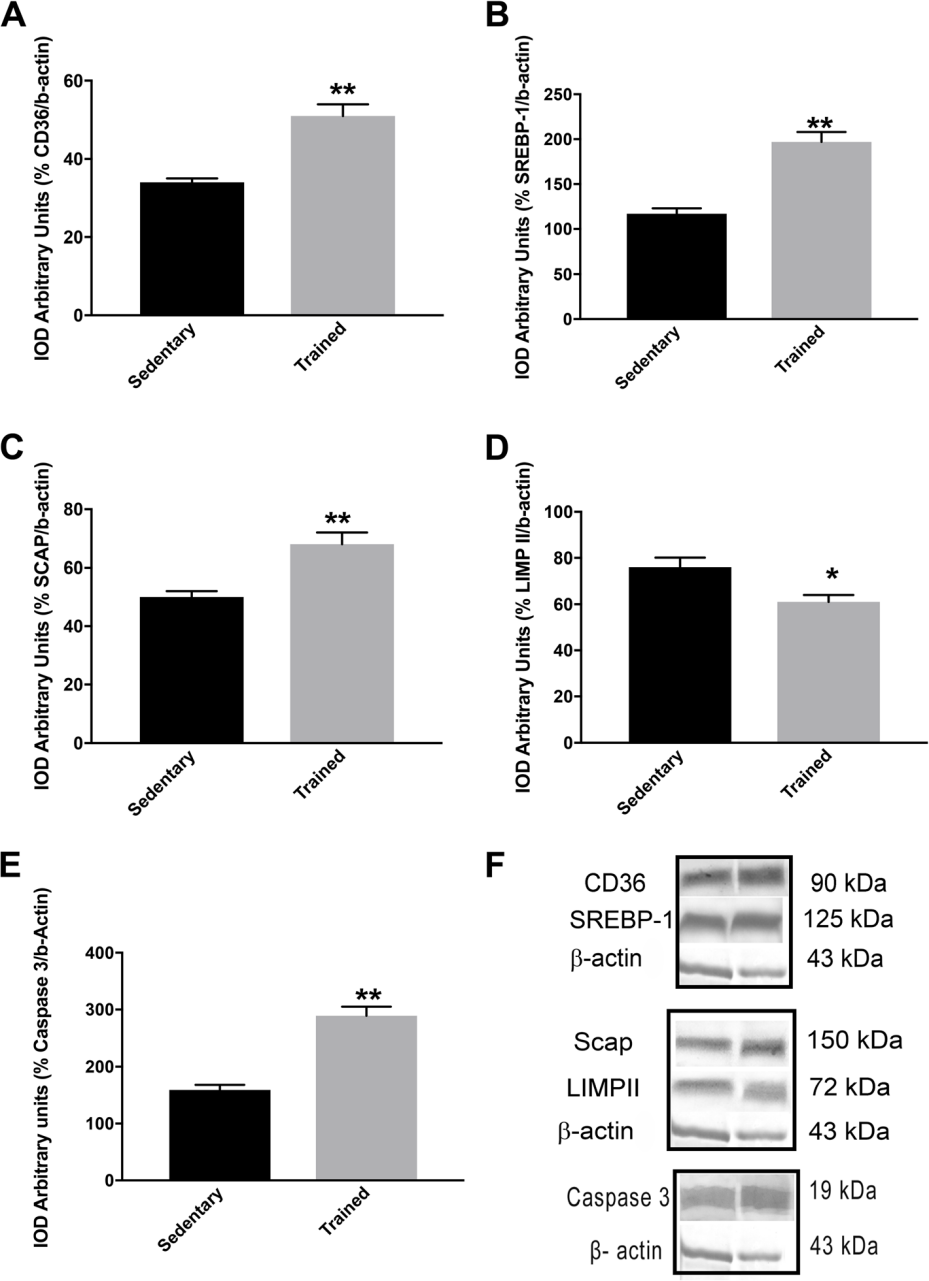
Changes in protein expression (in arbitrary units): (**a**) CD36, (**b**) SREBP-1, (**c**) SCAP, (**d**) LIMPII, (**e**) caspase-3, (**f**) CD-36, SREBP-1, β-actin, SCAP, LIMP II, and caspase-3 Western Blotting analysis. Values are expressed as mean ± SEM (*n* = 10); *p < 0,05, **p < 0,001, ***p < 0,0001

Interestingly, as shown in Fig. 3d, the level of LIMPII expression decreased in the prostate of trained rats relative to observations in sedentary animals. In contrast, physical exercise significantly increased CD36 expression (Fig. 3a). Additionally, expression of SCAP was significantly higher in trained rats compare to untrained rats (Fig. 3c). It was also observed that physical exercise induced expression of SREBP-1 in the rats submitted to resistance training (Fig. 3b). Similarly, levels of caspase-3 were higher in the trained rats compared to untrained rats (1.85-fold increase versus sedentary, *p* = 0.01; Fig. 3e).

## Discussion

The main findings of the present investigation were as follows: a) physical training is correlated with increased food consumption and energy intake, and decreased weight gain and FE; b) training decreases cholesterol content and increases the lipid fraction in blood plasma; c) an increase in the free testosterone and level of IGF-1 plasma levels are observed following resistance training; d) training upregulates expression of CD36, SCAP, and SREBP-1, while downregulating LIMPII expression; and e) caspase-3 and Bax indices increase following resistance training. Collectively, these data indicated that resistance training leads to increased lipid content in plasma and in the prostate. The results obtained in this study suggest that the development of insulin resistance and obesity may be prevented by improving FE along with greater food and energy intake, and this can be achieved with resistance training. Corroborating these results with those reported in literature, it was previously inferred that resistance training increases caloric expenditure but does not reduce plasma glucose levels [28].

To investigate the molecular events associated with physical exercise, we analyzed the lipid profile in the prostate and in plasma. Our study demonstrated that resistance training with a gradual increase in weight load has broad beneficial effects, such as decreased concentration of total cholesterol and increased HDL, LDL, and VLDL. The HDL/TG ratio is an indicator of the composition, amount, and density of LDL. A reduced HDL/TG ratio in the trained rats indicates smaller amounts of LDL cholesterol [29]. Exercise training appeared to exert a protective effect and enhanced the HDL/TG ratio. Animals showing a normal lipoprotein-lipid profile may require more exercise stimulation and energy expenditure coupled with significant reduction in bodyweight to further improve their lipid profile [30]. Resistance training also increases the level of serum TGs [31]. An increase in fatty acid oxidation is facilitated by heightened capacity for fatty acid uptake and their subsequent transport to mitochondria [32]. We propose that in the prostate, as in muscles, free fatty acid utilization, uptake, and/or storage as TG droplets or membrane constituents might be enhanced after physical exercise.

Prostate cells have been characterized to uptake fatty acid over glucose [33, 34] and activating the CD36 receptor, which is required for LDL oxidation, in PTEN/KO mice significantly impedes cancer progression. The current study found that CD36 is upregulated in the rat prostate following exercise. Similarly, triglyceride levels and the ratio of triglycerides to HDL is also higher in animals after resistance training, suggesting that the increase in CD36 expression is unlikely to be a compensatory mechanism in response to lower blood triglyceride levels. CD36 receptors bind LDL, HDL, and VLDL in the translocation of fatty acid, and are scavenger receptors that mediate apoptotic cell phagocytosis, as well as the recognition and internalization of oxidized lipoproteins and free fatty acids. CD36 is the defining member of a small family, which includes LIMPII and scavenger receptor B1 (SR-BI) in humans. In the prostate, except for LIMPII, all lipid receptors that regulate selective uptake of lipids (free fatty acids, cholesterol), lipoproteins (oxidized LDL, HDL, VLDL), and other molecules from the extracellular matrix, such as collagen and thrombospondin [35], were overexpressed after physical exercise. We showed that physical exercise results in an increase in CD36 and caspase-3 indices, and a decrease in LIMPII expression. CD36 transduces its signals through kinase p38 mitogen-activated protein kinase (MAPK) and c-Jun N-terminal kinase, both critical for apoptosis-mediated inhibition of angiogenesis by TSP-1 [36]. TSP-1 induces apoptosis of epithelial cell via the Fyn-dependent pathway involving activation of caspase-3-like proteases and p38 MAPK [37, 38]. A recent study conducted by our group reported that resistance training efficiently modulates cellular homoeostasis by reducing cell proliferation and increasing apoptosis [6], confirming that apoptosis is promoted by physical exercise.

As well as the CD36 protein, LIMPII binding to adhesive glycoprotein TSP-1 may affect platelet adhesion, angiogenesis, and apoptotic cells internalization. Exercise may reduce LIMPII expression by modulating protein trafficking and possibly reducing cholesterol accumulation in prostate cells. LIMPII regulates endosomal biogenesis and alters prostate cells, and investigating these biomarkers may be a novel method to aid in the diagnosis and prognosis of prostate cancer [39]. Recently, it has been shown that many lysosomal membrane proteins undergo proteolysis, which is mainly mediated by cathepsin proteases like cathepsin-F, indicating lysosomal LIMPII as substrate of cathepsin-F [40]. However, in this study, an increased expression of CD36 and decreased expression of LIMPII was observed. Overexpression of LIMPII impairs trafficking out of affected compartments, and this process is closely associated with cholesterol accumulation, although the nature of this correlation is not completely understood [41].

In line with cholesterol reduction, physical exercise increased SREBP-1 and, consequently, SCAP expression. The SREBP-SCAP-INSIG complex is stabilized by cholesterol. When steroid concentration is low, the SREBP-SCAP complex is released from INSIG, and localizes to the Golgi complex where SREBP-SCAP is proteolytically cleaved. The SREBP fragment is then translocated to the nucleus where it interacts with the transcriptional start sites of genes involved in lipid synthesis, lipid receptors, and fatty acid and cholesterol metabolism [10, 42]. As shown by Suh et al. [28], mature SREBP-1 represses AR functions, which inhibits the expression of AR-target genes and androgen-dependent growth of prostate cancer cells. These findings suggest that SREBP-1 may play an important role in cell growth as well as in the control of lipogenesis in prostate cancer cells. Studies in vivo and in vitro suggest that cholesterol synthesis induction by the AKT/mTORC1/SREBP pathway contribute to cell growth [43]. It is possible that the lipogenic action of SREBP-1 was reduced by the catabolic effect of physical exercise, increasing free radicals and, consequently, fatty acids oxidation via AMPK. AMPK activation restricts anabolic pathways, including lipogenesis, and represses cells growth and induces apoptosis [44]. Therefore, we suggest that physical exercise might inhibit SREBP-1 mediates lipogenesis through AMPK activation.

In the present study, we observed a significant increase in the concentration of free testosterone and IGF-1 in rats subjected to a 13 weeks of resistance training. Prostatic changes may be due to dysregulation of free testosterone and IGF-1, which are inducers of cell-cycle progression and apoptosis inhibitors [45, 46]. In addition, there is no relationship between the expression of prostate cancer markers and growth factors [47, 48], suggesting that increased IGF-1 and growth hormone may not compromise the progression and status of prostate diseases. We found that the increases in plasma free testosterone and plasma IGF-1 concentrations in rats undergoing physical training did not induce prostate cell growth, but instead promoted the expression of the apoptotic factors caspase-3 and Bax in prostate epithelial cells.

The study of prostatic responses to physical resistance training is relatively innovative and can relate negative pathological growth effects to lifestyle choices related to exercise. Relating the different types of physical training to anaerobic and aerobic energy metabolism with prostatic modulations may reveal the direct effects of activity on cellular apoptosis and lipid metabolism in cancer. There are some notable limitations to this study. Firstly, the animals in our study were exposed to 13 weeks of resistance training, though it is possible that other exercise protocols could yield improved results related to lipid metabolism and overall prostatic health. Considering this, it is important to investigate if other durations and forms of exercise, such as aerobic or concurrent training, produce findings comparable to those described in this study and if there is an optimal exercise regimen for improving prostate cancer risk and outcomes. Secondly, the molecular pathways in the prostate associated with resistance exercise need to be identified and this study did not explicitly evaluate the mechanisms of exercise-related changes to prostate health. As previously discussed, physical training increased apoptosis in a manner than may impact the epithelium, and the mechanisms underlying elevated apoptotic rates require further investigation. Future research should be conducted to further understand of how physical exercise may impact prostate health at the molecular level.

## Conclusions

It can be concluded that physical resistance training is able to mobilize energy substrates for efficient usage and this exercise also modulates an organism’s lipid profile, reflecting changes in the prostate microenvironment. Resistance training can be a valuable tool to improve the entry of fatty acids into prostatic epithelial cells, thereby increasing insulin sensitivity and regulating cell maintenance and development. As the pathways in these processes overlap and their details have yet to be fully elucidated, there is precedent for future studies in animal models of lipid pathways and prostatic changes associated with resistance training and other types of exercise.

## Data Availability

The datasets generated during and/or analyzed during the current study are available from the corresponding author on reasonable request.

## Abbreviations

AKT: Protein Kinase B
AR: Androgen receptor
Bax: Bcl-2 associated X protein
Bcl-2: B-cell Lymphoma 2
BSA: Bovine Serum Albumin
CD36: Cluster of differentiation 36
FE: Feed efficiency
HDL: High-density lipoprotein
IGF-1: Insulin-like growth factor-1
LDL: Low-density lipoprotein
LIMPII: Lysosome membrane protein
MAPK: Mitogen-activated protein kinase
mTORC1: Mammalian Target of Rapamycin Complex-1
PTEN: Prime Time Entertainment Network
SCAP: Sterol regulatory element-binding protein cleavage-activating protein
SREBP-1: Sterol regulatory element binding protein-1
TEI: Total energy consumption
TGs: Triglycerides
TSP-1: Thrombospondin 1
VLDL: Very low-density lipoprotein
